# Colovaginal Fistula in a Patient Without Diverticular Disease or Prior Hysterectomy: A Case Report

**DOI:** 10.7759/cureus.114057

**Published:** 2026-08-06

**Authors:** Sulagna Sensarma, MacKenzie Cole, Rohit Mantha, Shelby Wallace, Zachary Harwood

**Affiliations:** 1 Surgery, Obstetrics and Gynecology, Lincoln Memorial University DeBusk College of Osteopathic Medicine, Harrogate, USA; 2 Internal Medicine, HCA Florida Brandon Hospital, Brandon, USA

**Keywords:** colovaginal fistula, low anterior resection, pelvic adhesions, robotic-assisted surgery, sigmoidectomy

## Abstract

Colovaginal fistulas are abnormal communications between the colon and vagina that typically present with the passage of flatus or fecal material through the vagina and usually require surgical repair. Most cases occur in association with diverticular disease or a prior hysterectomy; occurrence in the absence of these risk factors is uncommon and may delay diagnosis. We present the case of a 69-year-old woman with a colovaginal fistula despite the absence of diverticular disease and a history of prior hysterectomy. She underwent successful multidisciplinary surgical management involving Urology, Gynecology, and General Surgery, followed by a favorable postoperative recovery. This report highlights the importance of considering colovaginal fistula in elderly patients presenting with feculent vaginal discharge, even in the absence of traditional risk factors, and demonstrates the value of coordinated multidisciplinary management in atypical presentations.

## Introduction

Colovaginal fistulas are a rare but distressing condition characterized by an abnormal communication between the colon and vagina. Unlike rectovaginal fistulas, which arise between the rectum and vagina, colovaginal fistulas most commonly develop secondary to diverticular disease and are frequently associated with a history of prior hysterectomy [1]. Patients without these classic risk factors are uncommon, which may delay clinical recognition and diagnosis. Reporting atypical presentations is therefore important for increasing clinician awareness and broadening the differential diagnosis in patients with suggestive symptoms.

Patients typically present in their sixth or seventh decade of life with the passage of flatus or fecal material through the vagina, malodorous vaginal discharge, recurrent vaginal infections, or abdominal pain [2]. Diagnosis is established through clinical evaluation and contrast imaging studies, including vaginography [3]. Definitive management involves surgical resection of the affected bowel segment with primary anastomosis and repair of the vaginal defect, with reported high success rates [4]. We report a case of colovaginal fistula in a patient without diverticular disease or prior hysterectomy, highlighting the importance of considering this diagnosis even in the absence of these classic predisposing conditions.

## Case presentation

A 69-year-old female, G1P1001, with a medical history of hyperlipidemia, chronic obstructive pulmonary disease (COPD), and gastroesophageal reflux disease (GERD), and a surgical history of appendectomy, one forceps-assisted vaginal delivery, and tubal ligation, presented as an inpatient with several months of feculent vaginal discharge, accompanied by dysuria and anal pain beginning in the fall of 2025. Physical examination revealed an opening along the left upper vaginal wall near the cervix, with loose yellow stool freely draining into the vaginal canal, raising concern for a colovaginal fistula. Gynecology, Infectious Disease, and Colorectal Surgery were consulted. Colorectal Surgery recommended an elective surgical resection with low anterior resection (LAR). The patient was discharged home in stable condition, with outpatient follow-up arranged through General Surgery at Clark Regional Medical Center.

**Figure 1:**
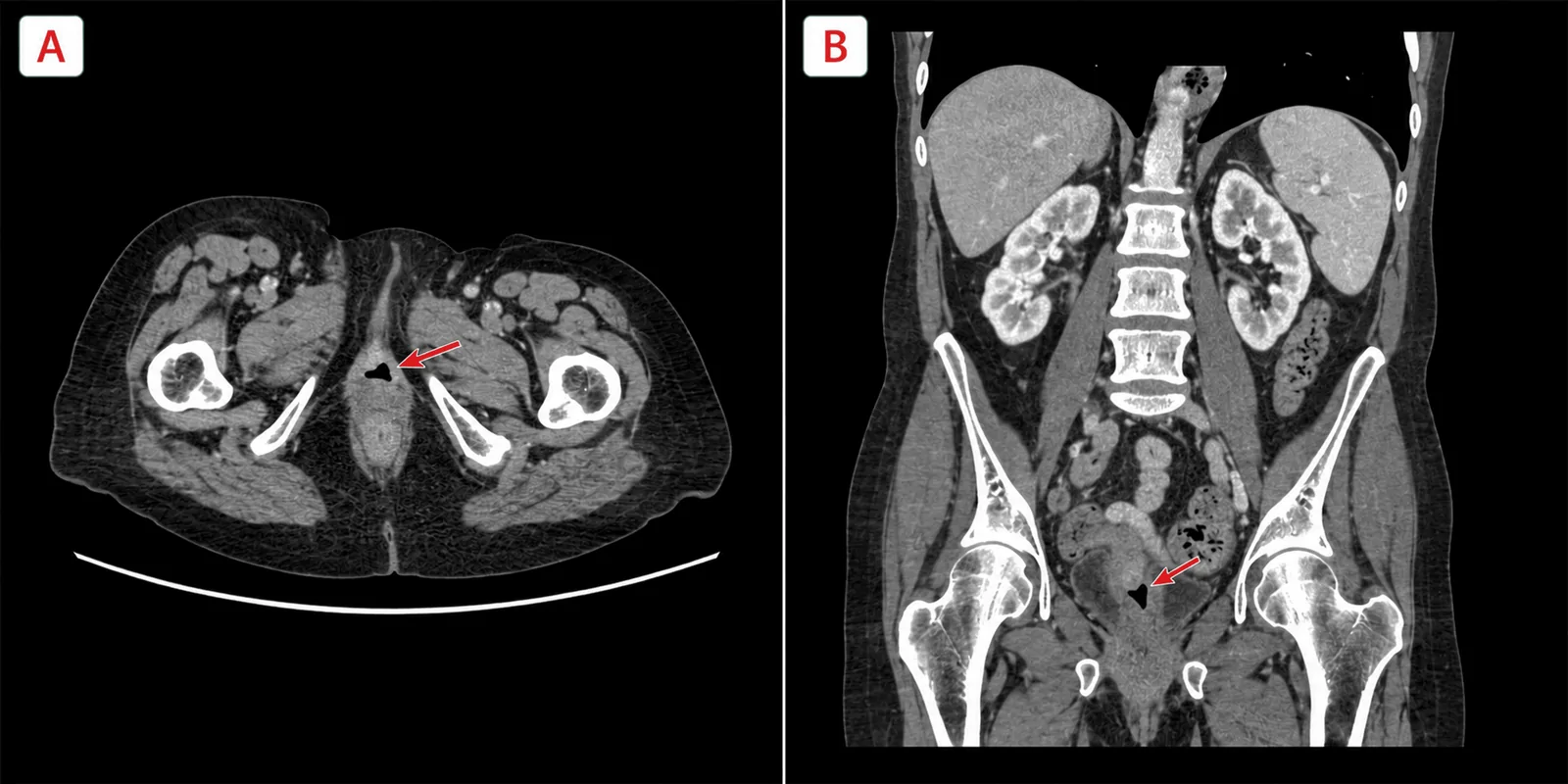
CT abdomen and pelvis CT demonstrating air within the vaginal vault (red arrows) on axial (A) and coronal (B) images, consistent with rectovaginal fistula formation, obtained one month after symptom onset CT: computed tomography

During outpatient follow-up, repeat CT imaging demonstrated air within the vaginal vault, a finding highly concerning for a colovaginal fistula (Figure 1). Colonoscopy revealed benign colonic polyps without evidence of malignancy, inflammatory bowel disease, or an identifiable intraluminal source of the fistula. Gynecologic reevaluation demonstrated severe vulvar and perirectal irritation secondary to continuous fecal leakage. Based on these findings, operative intervention was scheduled.

The patient subsequently underwent multidisciplinary surgical management involving Urology, Gynecology, and General Surgery. Urology initially placed bilateral ureteral stents to reduce the risk of ureteral injury. Gynecology then performed a transvaginal closure of the vaginal defect associated with the fistula and a right laparoscopic oophorectomy. The right ovary was markedly enlarged because of a known ovarian cyst and obstructed visualization of the surgical field, necessitating its removal. Granulation tissue surrounding the vaginal opening of the fistula was excised, the vaginal mucosa was closed, and topical estradiol cream was applied. General Surgery subsequently performed a robotic-assisted laparoscopic low anterior resection with takedown of the colovaginal fistula. A sigmoidectomy and upper proctectomy were completed, followed by excision of the fistulous tract between the sigmoid colon and vagina. A colorectal anastomosis was then created, and a surgical drain was placed.

**Figure 2:**
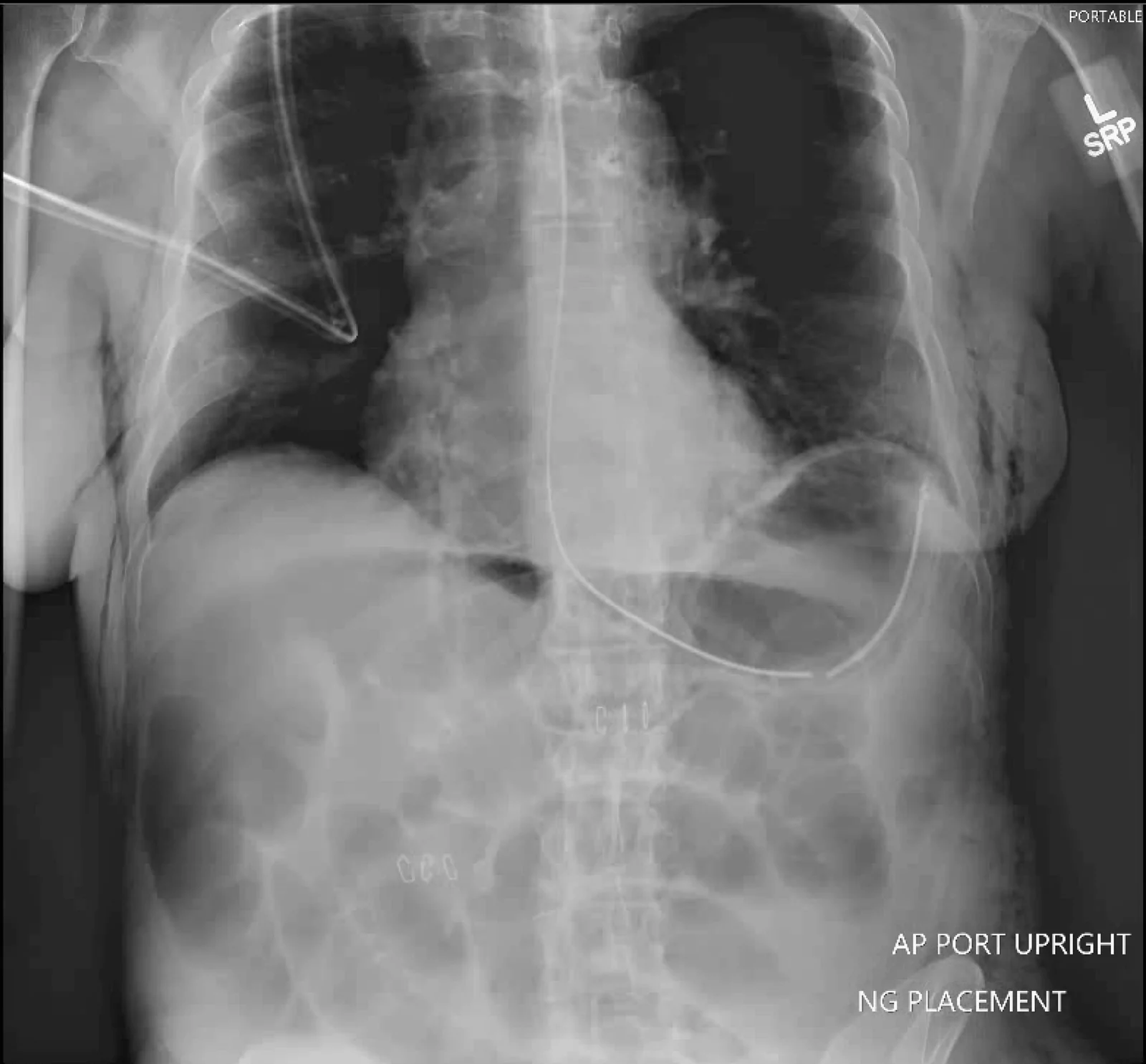
Abdominal radiograph The image demonstrates diffuse gaseous bowel distention consistent with postoperative ileus two months after the onset of symptoms

**Figure 3:**
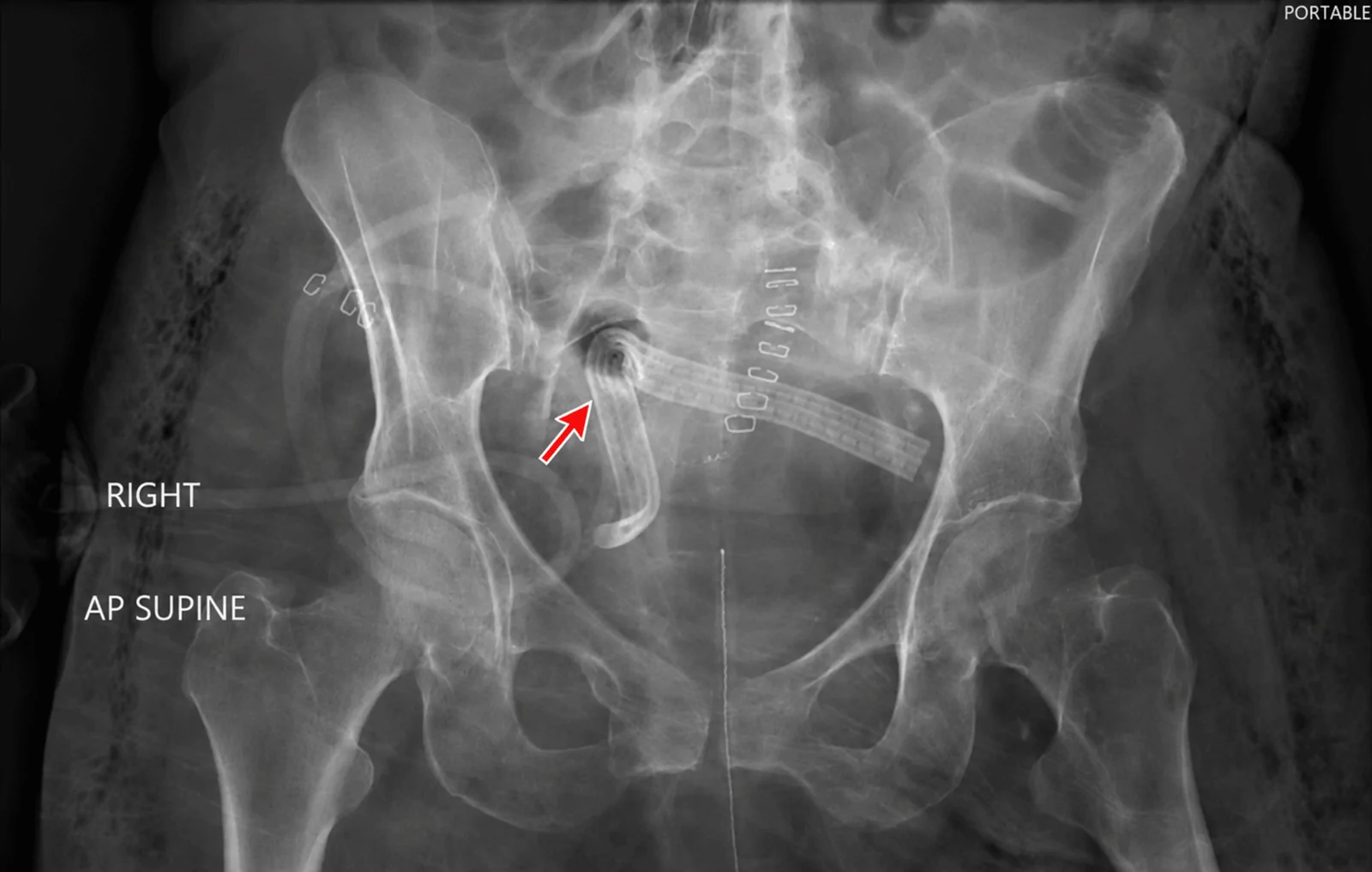
Portable abdominal radiograph The image demonstrates postoperative pelvic drain placement (red arrow) following robotic-assisted low anterior resection and fistula takedown two months after the onset of symptoms

Postoperatively, the patient remained in the medical-surgical unit for bowel rest and monitoring for six days. Vital signs remained stable throughout her stay. Laboratory studies demonstrated a mild postoperative elevation in the white blood cell count of 12.91 ×10³/µL (reference range: 4.0-11.0 ×10³/µL) and persistent anemia with hemoglobin values ranging from 8.7 to 9.5 g/dL (reference range: 12.0-16.0 g/dL) (Table 1). She also developed mild hypokalemia (nadir potassium 3.2 mmol/L) and elevated serum bicarbonate during nasogastric decompression, findings consistent with contraction metabolic alkalosis secondary to gastric fluid losses. Abdominal radiography demonstrated diffuse ileus, which was managed conservatively (Figure 2). The ileus resolved following return of flatus and bowel function. A nasogastric tube clamp trial was successful, and the tube was subsequently removed. The patient tolerated advancement to oral intake and was discharged home on postoperative day six in stable condition with a JP drain in place (Figure 3). Outpatient follow-up with General Surgery and Gynecology was arranged.

**Table 1:** Pertinent laboratory values obtained during the patient’s hospitalization Abnormal values are indicated as high (H) or low (L)

Laboratory parameter	Nov 18 (baseline)	Nov 21 (preoperative)	Nov 23 (postoperative)	Reference range
White blood cells	12.91×10³/µL (H)	9.36 ×10³/µL	6.96 ×10³/µL	4.0-11.0 ×10³/µL
Red blood cells	3.12 ×10⁶/µL (L)	2.81 ×10⁶/µL (L)	2.79 ×10⁶/µL (L)	3.8–5.1 ×10⁶/µL
Hemoglobin	9.5 g/dL (L)	8.7 g/dL (L)	8.8 g/dL (L)	12.0–16.0 g/dL
Hematocrit	29.1% (L)	27.3% (L)	26.4% (L)	36–46%
Sodium	139 mEq/L	142 mEq/L	139 mEq/L	135–145 mEq/L
Potassium	4.3 mEq/L	3.2 mEq/L (L)	3.2 mEq/L (L)	3.5–5.0 mEq/L
Carbon dioxide	27 mEq/L	34 mEq/L (H)	31 mEq/L (H)	22–29 mEq/L
Blood urea nitrogen	8 mg/dL	19 mg/dL (H)	7 mg/dL	7–20 mg/dL
Creatinine	0.8 mg/dL	0.9 mg/dL	0.9 mg/dL	0.6–1.2 mg/dL

## Discussion

This case represents an uncommon presentation of a colovaginal fistula occurring in the absence of the two most frequently associated risk factors: diverticular disease and prior hysterectomy. In typical cases, fistula formation is attributed to inflammatory processes from diverticulitis, in which a diverticular abscess or phlegmon erodes into the vaginal wall, establishing a pathological communication between the colon and vagina [2]. Similarly, hysterectomy has been shown to predispose patients to fistula development by eliminating the uterus as an anatomic barrier and leaving the vaginal cuff susceptible to direct injury and subsequent fistulization [5]. Although other etiologies, such as inflammatory bowel disease, radiation exposure, malignancy, and complications from colorectal surgery, have been described, their relative incidence remains low [5]. The absence of these established risk factors in our patient suggests that alternative mechanisms may contribute to fistula formation and highlights the need for individualized evaluation and multidisciplinary management in atypical presentations.

Our patient's presentation was consistent with previously reported cases despite lacking the typical predisposing factors. Reported symptoms include dysuria, anal or pelvic pain, and, most characteristically, feculent vaginal discharge. Our patient's age was within the typical range reported for patients with colovaginal fistulas. Consistent with prior reports, the fistulous communication involved the left vaginal apex, a location described in approximately 90% of cases [2]. Surgical management likewise reflected established treatment strategies, including resection of the diseased colonic segment with primary colorectal anastomosis and repair of the vaginal defect. Although the patient's postoperative course was complicated by a transient ileus requiring nasogastric decompression, bowel function recovered with conservative management, resulting in a total hospital stay comparable to that reported in the literature [6].

The distinguishing feature of this case was the absence of classic risk factors. Colonoscopic evaluation revealed no evidence of diverticular disease, with only benign polyps identified. This is in contrast to diverticulitis, which remains the most frequently reported etiology in the literature. Additionally, unlike the typical hysterectomy-associated colovaginal fistula, our patient retained her uterus and ovaries. Approximately 50% of hysterectomies are performed concurrently with bilateral salpingo-oophorectomy [7], with prior studies reporting a high prevalence of hysterectomy among patients with colovaginal fistulas. In the absence of this risk factor, the identification of an enlarged ovary complicated the procedure intraoperatively, necessitating additional gynecologic intervention.

Although the precise etiology remains unclear, multiple surgical, obstetric, and physiologic factors may have contributed. The patient's history of appendectomy and tubal ligation may have indirectly predisposed her to fistula development. Prior abdominal surgery is known to increase the risk of intra-abdominal adhesions, which can alter bowel mobility and pelvic anatomy [8]. While adhesions were not confirmed in this patient, postoperative anatomic changes represent one possible mechanism contributing to fistula formation. The patient's obstetric history is also noteworthy. Forceps-assisted vaginal delivery has been associated with injury to the vaginal wall and rectovaginal septum, potentially weakening these structures and increasing susceptibility to fistula formation later in life [9]. These effects may be compounded by age-related tissue atrophy and chronic hypoestrogenism, both of which are known to diminish tissue resilience, impair healing, and weaken pelvic floor support structures [10].

Taken together, we hypothesize that surgical and obstetric events, along with physiologic aging, may have interacted to produce an environment favoring fistula formation. Age-related changes in tissue integrity and vascularity may further contribute to impaired healing and susceptibility to fistula formation.

## Conclusions

This case report expands on the existing literature by demonstrating that colovaginal fistulas may occur in patients without the two most commonly recognized risk factors: diverticular disease and prior hysterectomy. Reliance solely on traditional predictors may lead to delayed diagnosis. Clinicians should maintain a high index of suspicion for colovaginal fistula in elderly patients presenting with fecal vaginal discharge, even in the absence of prior hysterectomy or diverticular disease. Early diagnosis is critical because these cases often require extensive multidisciplinary management. Atypical presentations warrant careful evaluation because management is often complex and multidisciplinary. Despite these challenges, postoperative recovery and overall outcomes may be comparable to those observed in patients with more typical presentations, highlighting that favorable outcomes can be achieved with prompt diagnosis, thorough operative planning, and multidisciplinary collaboration. This report reinforces the importance of clinical vigilance and prompt recognition to optimize patient outcomes.
